# Potential use of superparamagnetic iron oxide nanoparticles for *in vitro* and *in vivo* bioimaging of human myoblasts

**DOI:** 10.1038/s41598-018-22018-0

**Published:** 2018-02-27

**Authors:** Kamil R. Wierzbinski, Tomasz Szymanski, Natalia Rozwadowska, Jakub D. Rybka, Agnieszka Zimna, Tomasz Zalewski, Karolina Nowicka-Bauer, Agnieszka Malcher, Magdalena Nowaczyk, Michal Krupinski, Michal Fiedorowicz, Piotr Bogorodzki, Pawel Grieb, Michal Giersig, Maciej K. Kurpisz

**Affiliations:** 1 Institute of Human Genetics, Polish Academy of Sciences, Poznan, Poland; 2 Faculty of Chemistry, Adam Mickiewicz University, Poznan, Poland; 3 Wielkopolska Centre of Advanced Technologies, Adam Mickiewicz University, Poznan, Poland; 4 NanoBioMedical Centre, Adam Mickiewicz University, Poznan, Poland; 5 The Henryk Niewodniczanski Institute, Institute of Nuclear Physics Polish Academy of Sciences, Cracow, Poland; 6 Mossakowski Medical Research Centre, Polish Academy of Sciences, Warsaw, Poland; 7 Institute of Experimental Physics, Freie Universität Berlin, Berlin, Germany

## Abstract

Myocardial infarction (MI) is one of the most frequent causes of death in industrialized countries. Stem cells therapy seems to be very promising for regenerative medicine. Skeletal myoblasts transplantation into postinfarction scar has been shown to be effective in the failing heart but shows limitations such, e.g. cell retention and survival. We synthesized and investigated superparamagnetic iron oxide nanoparticles (SPIONs) as an agent for direct cell labeling, which can be used for stem cells imaging. High quality, monodisperse and biocompatible DMSA-coated SPIONs were obtained with thermal decomposition and subsequent ligand exchange reaction. SPIONs’ presence within myoblasts was confirmed by Prussian Blue staining and inductively coupled plasma mass spectrometry (ICP-MS). SPIONs’ influence on tested cells was studied by their proliferation, ageing, differentiation potential and ROS production. Cytotoxicity of obtained nanoparticles and myoblast associated apoptosis were also tested, as well as iron-related and coating-related genes expression. We examined SPIONs’ impact on overexpression of two pro-angiogenic factors introduced via myoblast electroporation method. Proposed SPION-labeling was sufficient to visualize firefly luciferase-modified and SPION-labeled cells with magnetic resonance imaging (MRI) combined with bioluminescence imaging (BLI) *in vivo*. The obtained results demonstrated a limited SPIONs’ influence on treated skeletal myoblasts, not interfering with basic cell functions.

## Introduction

The last two decades yielded numerous research attempts and concepts regarding the usage of nanoparticles in biomedicine. They were thoroughly examined for such applications as drug delivery vectors, contrast agents for imaging, administration of particular antigens in selected diseases (blood stream), controlled drug release and prevention of skin aging^1–3^. Moreover, they have been used as nanocarriers for vaccinations^4^. Nanoparticles were also tested in cancer therapy when using hyperthermic algorithm. Tissues loaded with superparamagnetic iron oxide nanoparticles (SPIONs) were treated with magnetic field which led to production of heat resulting in cell necrosis^5^. SPIONs, due to their magnetic properties, have a great potential in magnetic resonance imaging (MRI). MRI is a well-developed precise instrument in medicine that offers soft tissue contrast, without using ionizing radiation or potentially harmful radiotracers. This method can be used in diagnostics of various diseases including oncological pathologies^2^.

SPIONs can also be used as agents for direct cell labeling and thereby they may have a great potential in stem cells therapy. Stem cell therapy used for recovery of the myocardial muscle function after cardiac infarction is a very promising and thoroughly investigated concept throughout the recent years^6–8^. The basis of this therapy is the direct administration of stem cells to a vicinity of the postinfarction scar composed mainly of connective tissue. In simple terms, stem cells migrate along the scar and differentiate into myotubes. There were many preclinical and clinical trials conducted, employing various types of stem cells, i.e. adult stem cells like skeletal myoblasts^9^ and cardiac stem cells (CSCs)^10^ or bone marrow derived mesenchymal stem cells (MSCs)^11^.

Myoblasts are unipotent stem/progenitor cells of skeletal muscle tissue reservoir, which originate from muscle satellite cells activated by muscle injury or exercise. Myoblasts differentiate and fuse with each other creating polynucleated myotubes and muscle fibres^12^. Taking into account that myoblasts differentiate to contractile cells similar to cardiomyocytes with an ability to resist hypoxic conditions^13^, they seem to be promising candidates for stem cell therapy of postinfarction heart.

However, to date, the examined procedures showed, regardless of the cell types used, rather moderate efficacy in the pro-regeneration of postinfarction, ischemic heart^6,8^. These studies indicated major struggles to overcome present limitations in efficacy, low retention and cell engraftment^14,15^ as well as the low proportion of stem cells differentiated into the functional cardiomyocytes^16^. There are variety of approaches to diminish these negative setbacks. First, is the use of various cytokines or other biological factors to accommodate the microenvironment of postinfarcted heart, promoting neovascularization^17^ or cell myogenic differentiation^17–19^. Second, is the functional optimization of stem cells, such as stimulation of gap junctions formation in order to reduce arrhythmia^20^.

Another crucial aspect of stem cell therapy is the visualization and monitoring of stem cells after their administration. A plethora of tools were employed to address this problem – radionuclides e.g. [F-18]-fluoro-2-deoxy-D-glucose (^18^F-FDG) for PET^21^ imaging and indium-111-oxyquinoline (^111^In-oxine) for SPECT imaging^22^, superparamagnetic iron oxide nanoparticles (SPIONs)^23^ and gadolinium Gd^3+^^24^ for MRI imaging, reporter genes such as herpes simplex virus thymidine kinase (HSVtk)^25^ and firefly luciferase^26^.

Taking into consideration all the problems that may appear in stem cells therapies, such as monitoring of stem cells migration, their retention in target site and their viability, the application of SPIONs can be promising due to their unique properties, that may be helpful to improve currently existing protocols. However, there is also a need to carefully evaluate any potential side effects which nanoparticles may bring to the cells. Here, we present a simple method of synthesis hydrophilic, biocompatible superparamagnetic iron oxide nanoparticles and evaluation of their biological impact on isolated human skeletal myoblasts.

## Results

### TEM

**Figure 1:**
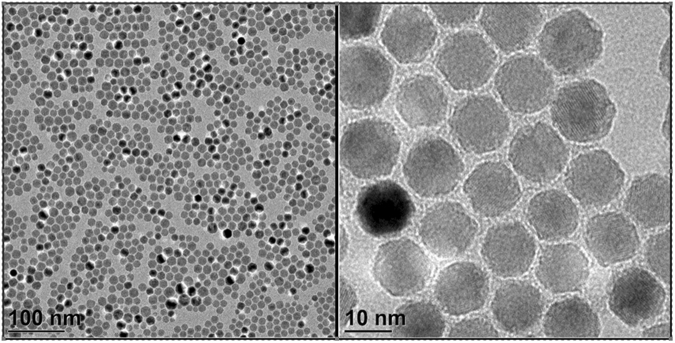
TEM images with nanoparticles synthesized in organic phase. Two different magnifications are presented 105000x and 810000x. Abbreviations: TEM – transmission electron microscope.

**Figure 2:**
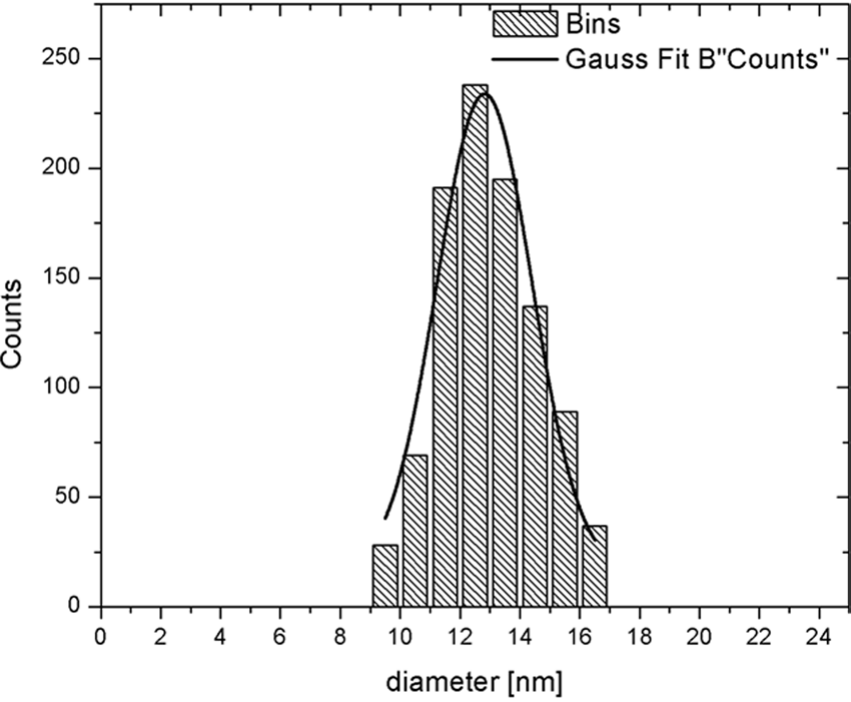
Nanoparticle size distribution determined with ImageJ software.

**Figure 3:**
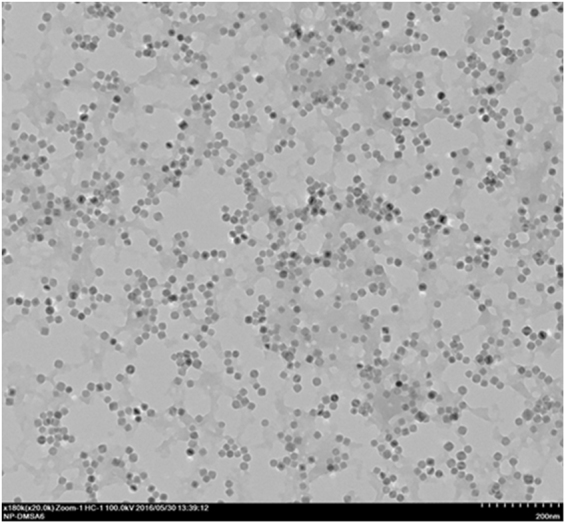
TEM image of DMSA coated nanoparticles. Scale bar indicates 200 nm. Abbreviations: TEM – transmission electron microscope; DMSA – meso-2,3-dimercaptosuccinic acid.

Transmission electron microscopy images (Fig. 1), show monodisperse nanoparticles with narrow size distribution (Figs 2 and 3) and well-rounded morphology. Good separation among nanoparticles is visible, due to hydrophobic repulsion forces between oleic acid moieties covering nanoparticles and electrostatic repulsion between negatively charged carboxyl groups from DMSA. Moreover, different facets of nanoparticles can be distinguished by different color saturation, which represents different plane of the crystal structure.

### FT-IR

**Figure 4:**
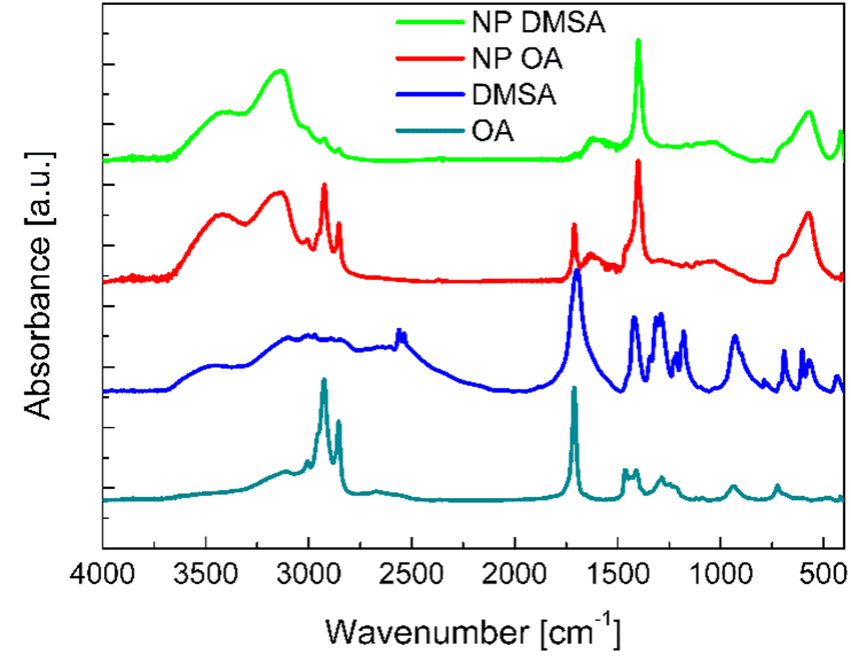
FT-IR plots, representing four spectra: azure – oleic acid, blue – DMSA, red – nanoparticles coated with oleic acid, green – nanoparticles coated with DMSA. Abbreviations: FT-IR – Fourier Transformed Infrared Spectroscopy; DMSA - meso-2,3-dimercaptosuccinic acid.

FTIR analysis showed peaks around 574 cm^−1^ and 623 cm^−1^ in NP OA and NP DMSA spectra. The peaks are attributed to the stretching vibrations from Fe-O and confirm the existence of nanoparticles with magnetite core^27^ (Fig. 4). There is also an interesting region for all spectra located between 1350 and 1750 cm^−1^. Sharp peaks at 1715 cm^−1^ in OA and 1700 cm^−1^ in DMSA spectrum are assigned to the symmetric stretching of a carbonyl group C=O^28,29^. Absorption decrease of carbonyl bands for free oleic acid is noticeable in comparison between NP OA and pure OA spectra. Reduction of absorption of the carbonyl group and the presence of two new peaks at 1400 cm^−1^ and 1630 cm^−1^ in NP OA spectra associated with symmetric and asymmetric stretching of carboxylate (COO^−^),^28–30^ indicates a covalent bond between oleic acid and iron oxide’s surface^27,29^. The similar decrease in the absorption band for the carbonyl and the presence of vibrations from carboxylate group are visible in spectrum for nanoparticles with DMSA (NP DMSA). Appearance of these peaks suggests that process of the ligand exchange was successful and DMSA is bound to the nanoparticles surface^28^. Broad bands at the region 3000–3600 cm^−1^ corresponding to the vibrations of the hydroxyl group (O-H) are also present in all spectra^31^. Sharp peaks from methylene group vibrations visible at spectra OA, NP OA and NP DMSA at 2854 cm^−1^ are assigned to the symmetric stretching and at 2924 cm^−1^ to the asymmetric stretching^32^. Appearance of weak bands of the C-H stretch group in the NP DMSA spectra, may be associated with presence of the residue of oleic acid on the nanoparticles surface after the ligand exchange^28^. Weak, sharp peaks at 2554 cm^−1^ and 2562 cm^-1^ observed in the spectrum for DMSA could be assigned to the vibrations of thiol groups (S-H)^31^. They are not visible in NP DMSA spectrum probably due to the oxidation of thiol groups into disulfide, which cannot be noticed in FTIR analysis because of low absorption of disulfide group^28^.

### Zeta Potential

**Figure 5:**
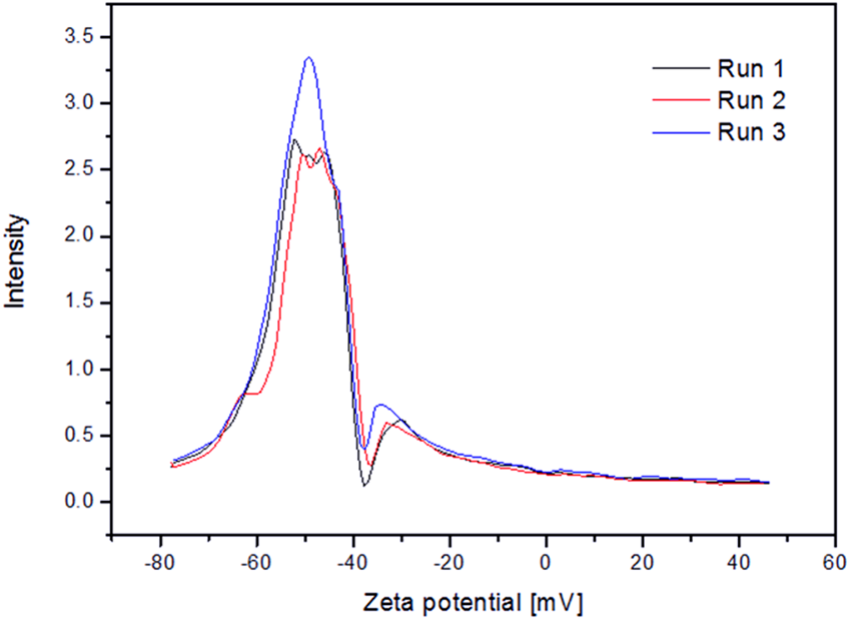
Zeta potential plot, 3 measurements are presented.

Zeta potential measurement was made for DMSA coated nanoparticles. Measurement indicates average ζ = −49,3 mV in water (Fig. 5). Such high value suggests strong negative charge at the surface of the nanoparticles, yielding high colloidal stability of nanoparticle suspension due to strong electrostatic repulsive forces between carboxyl groups. As a quality reference, correlation function was analyzed (not shown).

### SQUID and NMR relaxivity

**Figure 6:**
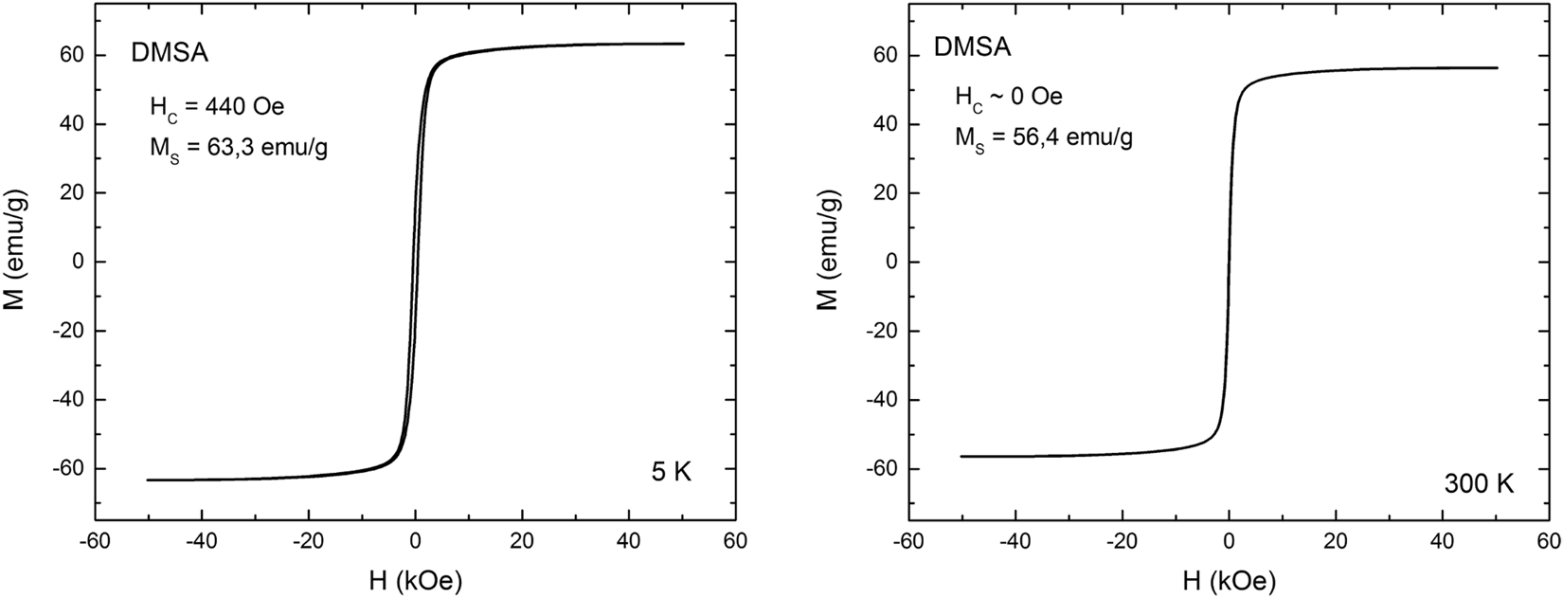
Hysteresis loops for 5 K and 300 K obtained by SQUID magnetometer.

**Figure 7:**
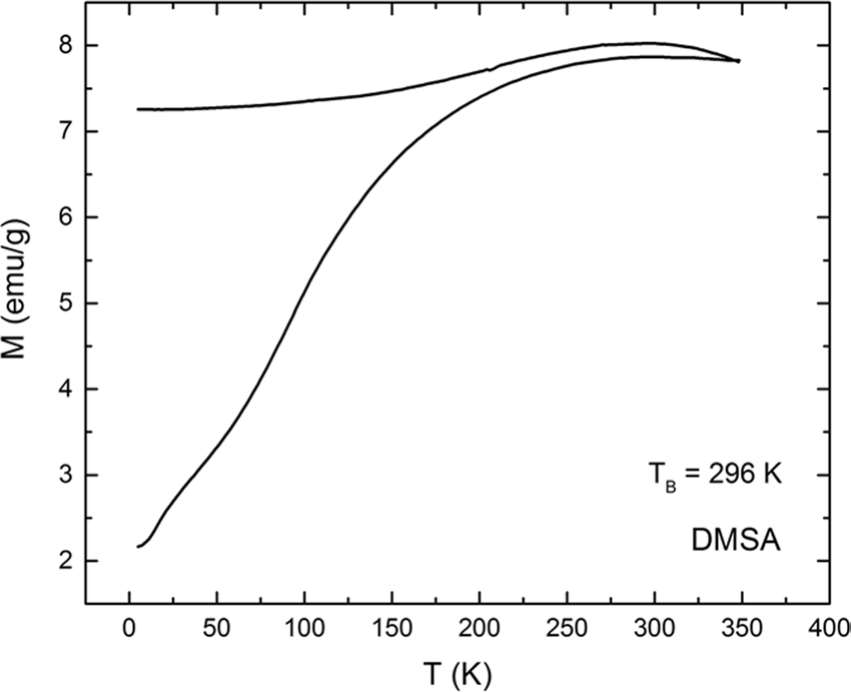
ZFC-FC plot, presenting blocking temperature of DMSA-coated nanoparticles.

**Figure 8:**
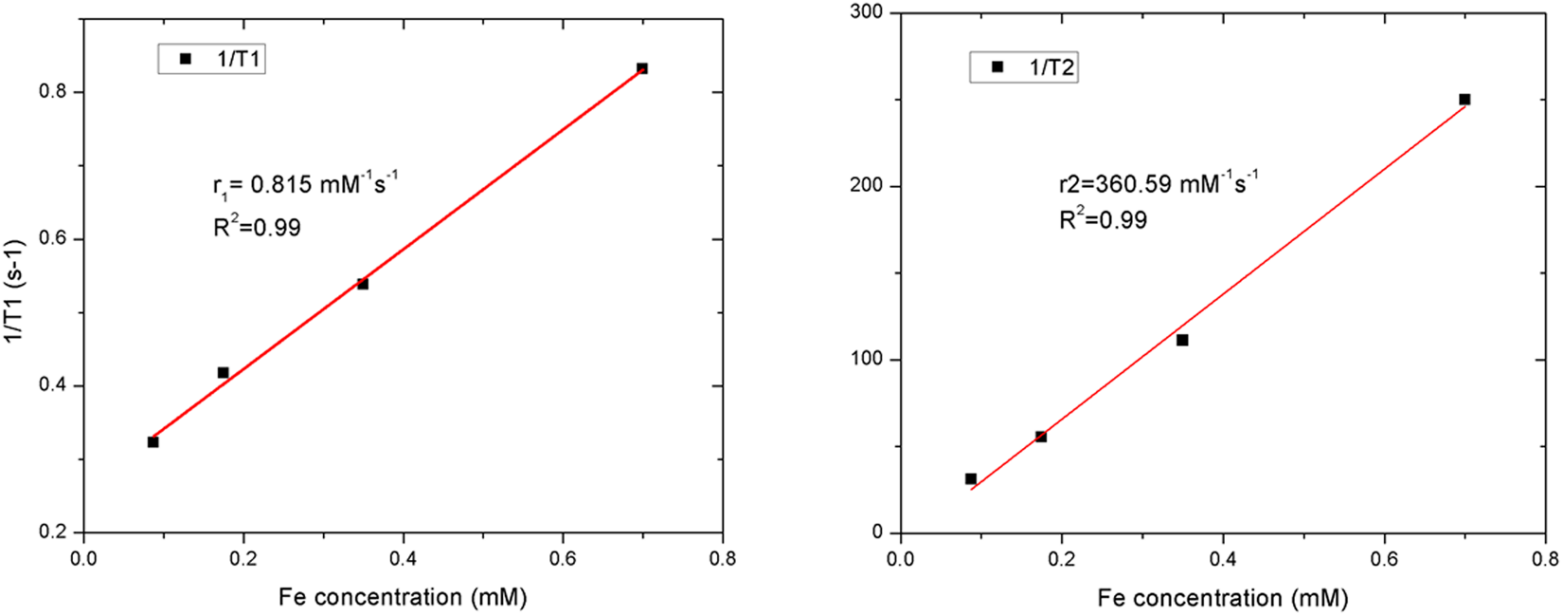
*T*_*1*_ and *T*_2_ relaxation time plots as a function of iron atoms concentration.

Hysteresis loops at 300 K shows H_c_ = 0 Oe, which means that there is no energy dissipated during repeated reversing of magnetization – defining the material as superparamagnetic. Nanoparticles also possess high value of saturation magnetization M_s_ = 56,4 emu/g, rendering them potentially very sensitive MRI contrast agent (Fig. 6). Blocking temperature obtained from ZFC-FC measurement is 296 K, which is near the room temperature (Fig. 7). Relaxivity studies of DMSA-coated nanoparticles showed a strong influence towards transverse relaxation process (*T*_2_ relaxation), r_2_ parameter value was 360 mM^−1^s^−1^, however, there was not observed a significant influence on *T*_1_ relaxation because experiments were conducted at high magnetic field (Fig. 8).

### Iron oxide nanoparticles cell labeling and SPIONs uptake evaluation

**Figure 9:**
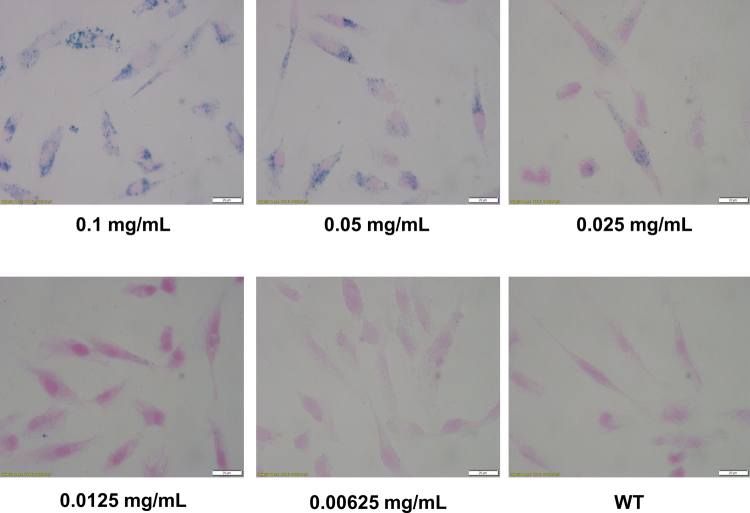
Prussian Blue staining of human myoblasts cultured in different concentrations of superparamagnetic iron oxide nanoparticles (SPIONs). Abbreviations: WT - myoblasts cultured in medium without Fe_3_O_4_-NPs. All images were taken in bright field setting with magnification 100x. Scale bars indicate 100 μm.

Prussian Blue staining of examined human myoblasts confirmed spontaneous uptake of SPIONs by cells and presence of iron oxide nanoparticles within labeled myoblasts accumulated through the whole cytosol. Fe_3_O_4_-NP labeling of cells shows difference in aggregation of nanoparticles, which is dependent from SPIONs concentration in culture medium. The higher concentration of SPIONs, the better visibility of Fe_3_O_4_-NPs aggregates in human myoblasts observed with Prussian Blue staining (Fig. 9). However, we did not observe any blue stained aggregates of iron oxide nanoparticle within myoblasts cultured with 0.00625 mg/mL concentration of SPIONs, so this group was not further tested in the following assays. In order to evaluate quantitatively our previous observations from the Prussian Blue staining experiments, we have measured cellular iron concentrations with mass spectrometry (Supplementary Figure S1). Examined cells showed almost exactly the same levels of cellular iron (Supplementary Figure S1C,D) as compared to standard curves in which where known amounts of iron were present (Supplementary Figure S1A,B). Hence, we can imply that virtually all nanoparticles which were given to the culture medium, were engulfed by myoblasts after 24 hours.

### Functional evaluation of human myoblasts labeled with SPIONs

**Figure 10:**
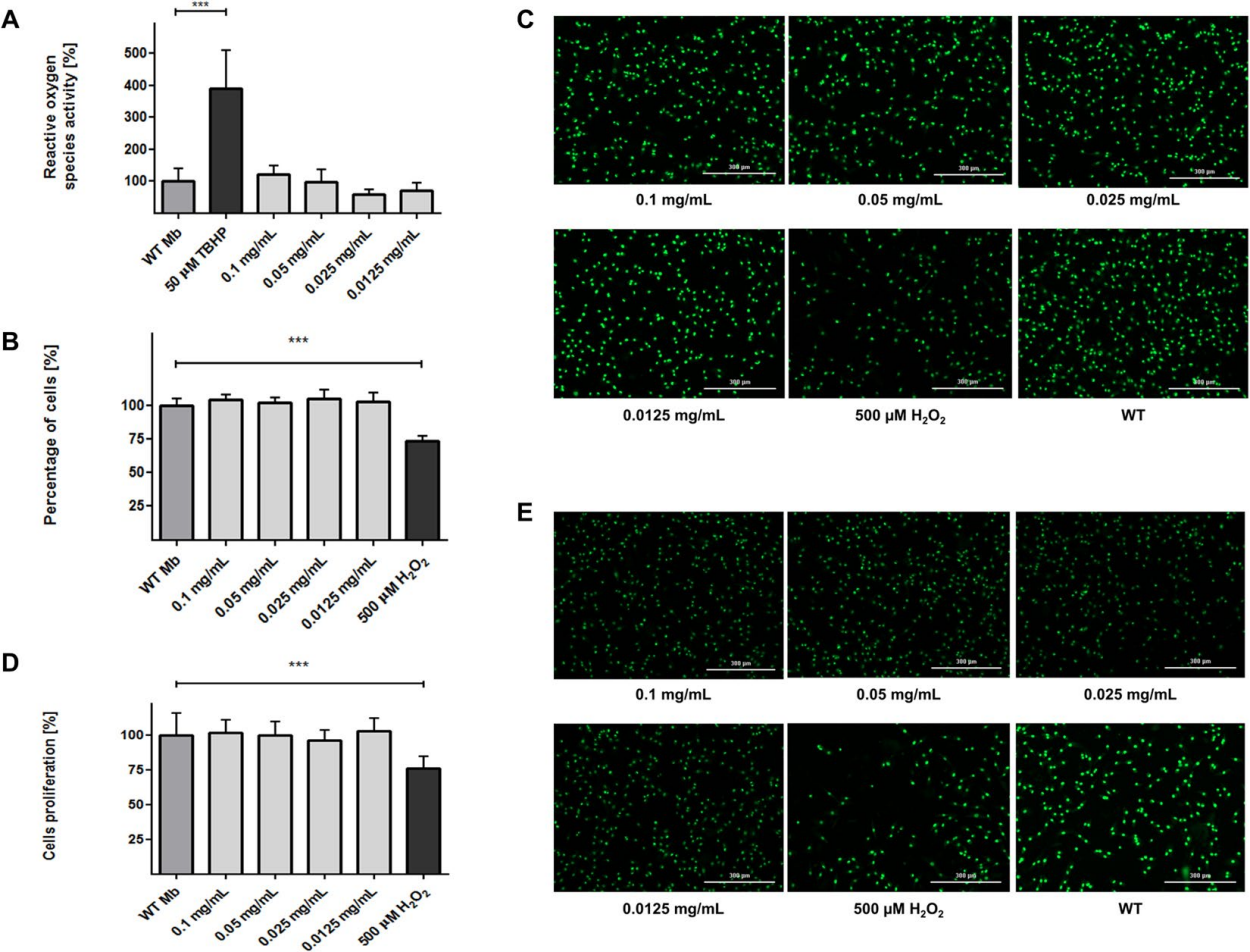
(**A**) Analysis of the reactive oxygen species activity in the studied populations of human myoblasts. The graph illustrates the relative fluorescence in tested cell populations comparing to the fluorescence of WT Mb. All cells were treated with 2′,7′-dichlorofluorescin diacetate. Positive control: 50 µM TBHP - myoblasts non-labeled with SPIONs, treated with 2′,7′-dichlorofluorescin diacetate and 50 µM tert-butyl Hydrogen Peroxide (TBHP). No significant differences between the populations under study were observed. (**B**,**C**) Evaluation of potential cytotoxicity of obtained SPIONs. The graph illustrates the relative fluorescence in tested cell populations comparing to the fluorescence of WT Mb (**B)**. No significant differences between the populations under study were observed. The images show fluorescent stained nuclei of only vivid cells in investigated groups (**C**). (**D**,**E**) Examination of impact of obtained nanoparticles on investigated cells proliferation ability. The graph illustrates the relative fluorescence in tested cell populations comparing to the fluorescence WT Mb (**D**). No significant differences between the populations under study were observed. The images illustrate fluorescent stained nuclei of only vivid cells in investigated samples 72 h after exposure of cells to nanoparticles (**E**). Abbreviations: WT Mb – myoblasts non-labeled with SPIONs. Asterisks indicate statistical significance (***p < 0.001). Scale bars indicate 300 µm.

Reactive oxygen species activity assay showed that there is no significance difference in ROS activity in WT myoblasts and in myoblasts labeled with different concentrations of Fe_3_O_4_-NPs. The highest level of fluorescence was observed in positive control (statistical significance p < 0.001) (Fig. 10A).

There were no statistically significant differences between untreated and labeled cells in cytotoxicity test (Fig. 10B,C). Proliferation assay performed 72 hours after exposure myoblasts to different concentrations of SPIONs in culture media demonstrated that there is also no impact of obtained nanoparticles on investigated cells proliferation ability (Fig. 10D,E).

Considering data obtained from Prussian Blue staining, reactive oxygen species activity assay, cytotoxicity and proliferation tests, we decided to perform next experiments with human myoblasts cultured in medium with 0.025 mg/mL concentration of SPIONs.

**Figure 11:**
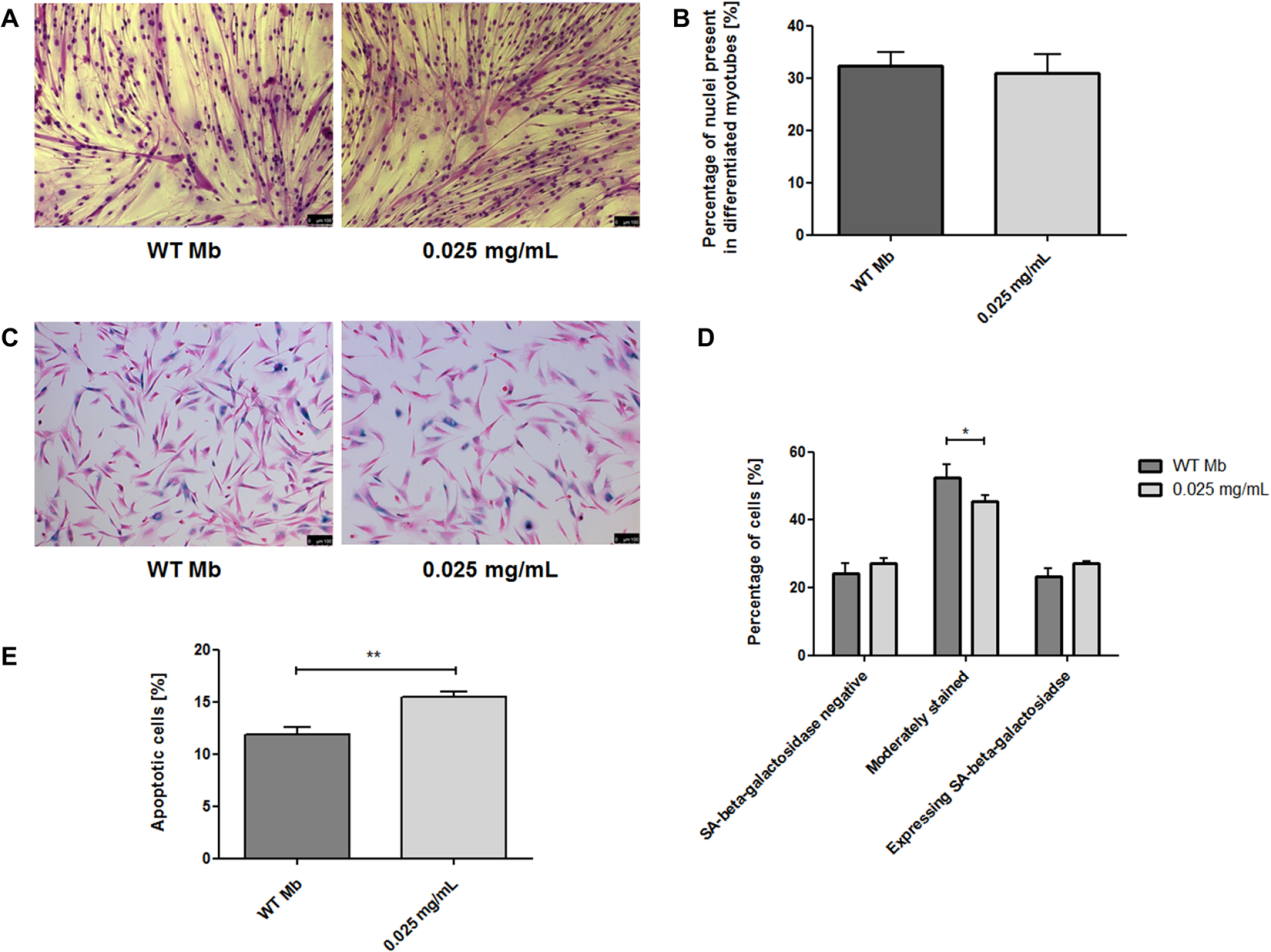
(**A**,**B**) Analysis of the differentiation potentials of human myoblasts. The images illustrate the potential of the Mb WT and labeled cell populations for differentiation into multinucleated myotubes (**A**). No significant differences between the populations under study were observed (**B**). (**C,D**) β-galactosidase staining assay. The images show Mb WT and labeled cells stained with SA-beta-galactosidase (**C**), Cell aging evaluation in myoblast populations under study (**D**). (**E**) Analysis of apoptosis in the studied populations of human myoblasts. Slightly increased cell apoptosis under normal *in vitro* cell culture conditions was observed in labeled cell population. Abbreviations: Mb WT – non-labeled myoblasts. Asterisks indicate statistical significance (*p < 0.05; **p < 0.01). Scale bars indicate 100 µm.

Analysis of the differentiation potential of human myoblasts enabled observations of microscopic images of WT myoblast and myoblasts with Fe_3_O_4_-NPs (Fig. 11A). Analysis of the myoblast differentiation potential into myotubes showed that there is no significant difference in number of nuclei present in differentiated myotubes of both WT and labeled populations of myoblasts (Fig. 11B).

Beta-galactosidase staining assay enabled observations with microscopic images of WT myoblast and myoblast labeled with Fe_3_O_4_-NPs (0.025 mg/mL) (Fig. 11C). Analysis of cell aging in beta-galactosidase assay showed significantly greater amount of WT myoblasts versus Fe_3_O_4_-NPs labeled in a group of matured cells (II - moderately stained) (p < 0.05). There are no significant differences between WT and Fe_3_O_4_-NPs labeled myoblasts in young and aged group of cells (I - SA-beta-galactosidase negative, III - expressing SA-beta-galactosidase), (Fig. 11D).

Analysis of apoptosis in WT and Fe_3_O_4_-NPs labeled myoblast populations showed moderately higher amount of apoptotic cells in Fe_3_O_4_-NPs group. This result indicates statistical significance (p < 0.01), (Fig. 11E).

### Real-time PCR – gene expression study

**Figure 12:**
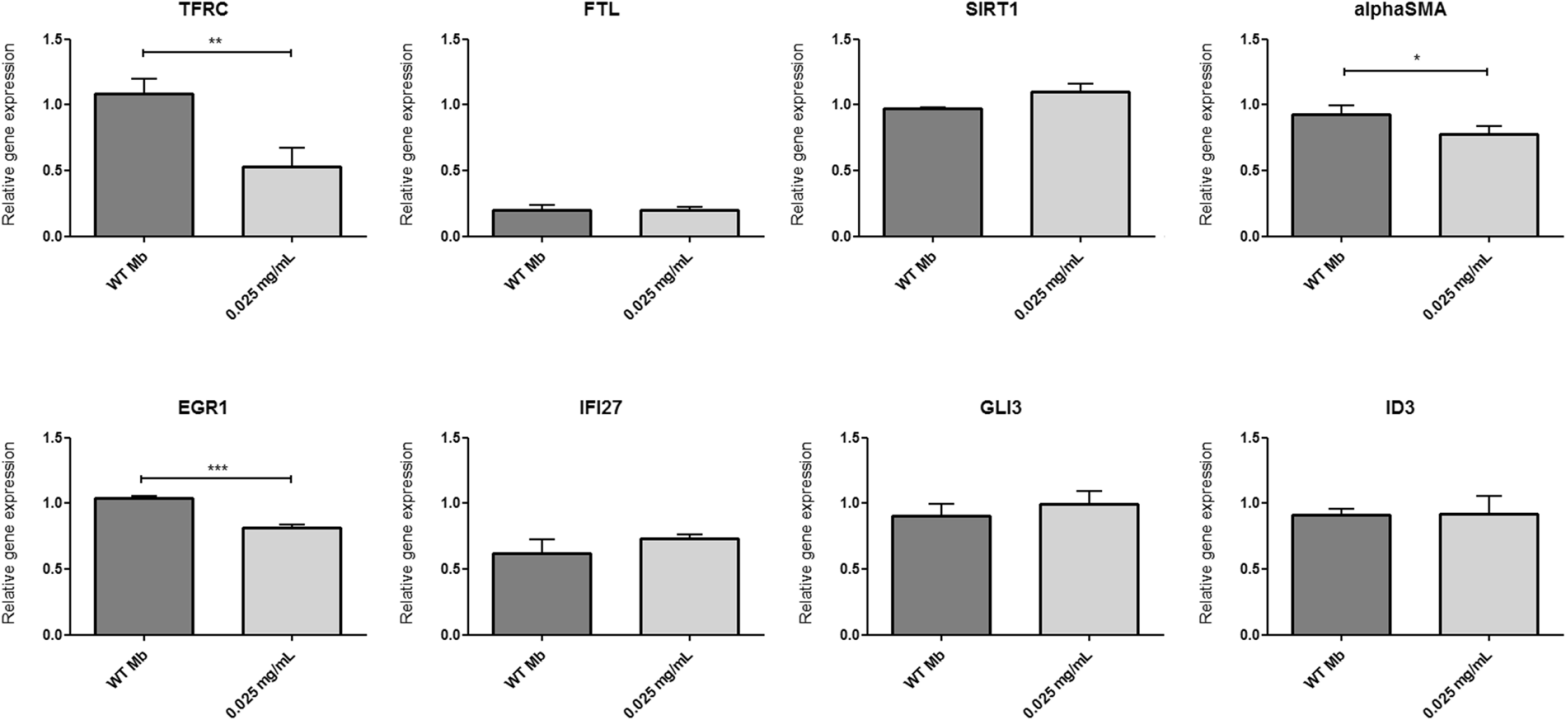
Expression of selected genes in the studied populations of human myoblasts evaluated by real-time qPCR. The relative expression of genes was normalized according to the expression of a housekeeping genes: *β-actin*, *HPRT1* and *GAPDH*. The data are presented as a relative mRNA fold. Asterisks indicate statistical significance (*p < 0.05; **p < 0.01; ***p < 0.001). Abbreviations: Mb WT – non-labeled myoblasts; *HPRT1* – hypoxanthine phosphoribosyltransferase 1; *GAPDH* – glyceraldehyde-3-phosphate dehydrogenase; *TFRC* – transferrin receptor 1; *FTL* – ferritin light chain; *SIRT1* – sirtuin 1; *alphaSMA* – alpha smooth muscle actin; *EGR1* – early growth response 1; *IFI27* – interferon alpha inducible protein 27; *GLI3* – GLI family zinc finger 3; *ID3* – inhibitor of DNA binding 3

Analysis of examined selected genes expression in studied populations of human myoblasts indicated significant differences in expression of some genes between WT and Fe_3_O_4_-NPs myoblasts (Fig. 12). Relative expression of *TFRC* was higher in WT myoblasts than for SPION-labeled sample. This result indicates statistical significance (p < 0.01). Relative expression of *alpha-SMA* gene was also higher in WT myoblasts with statistical significance (p < 0.05). Another difference was observed in relative expression of *EGR1* which again was higher in WT myoblasts than in SPION-labeled population and this also indicated statistical significance (p < 0.001). There were no statistically significant differences noted in relative expression of *FTL*, *SIRT1*, *IFI27*, *GLI3* and *ID3* genes between labeled and WT myoblasts.

### HUVEC angiogenesis test

**Figure 13:**
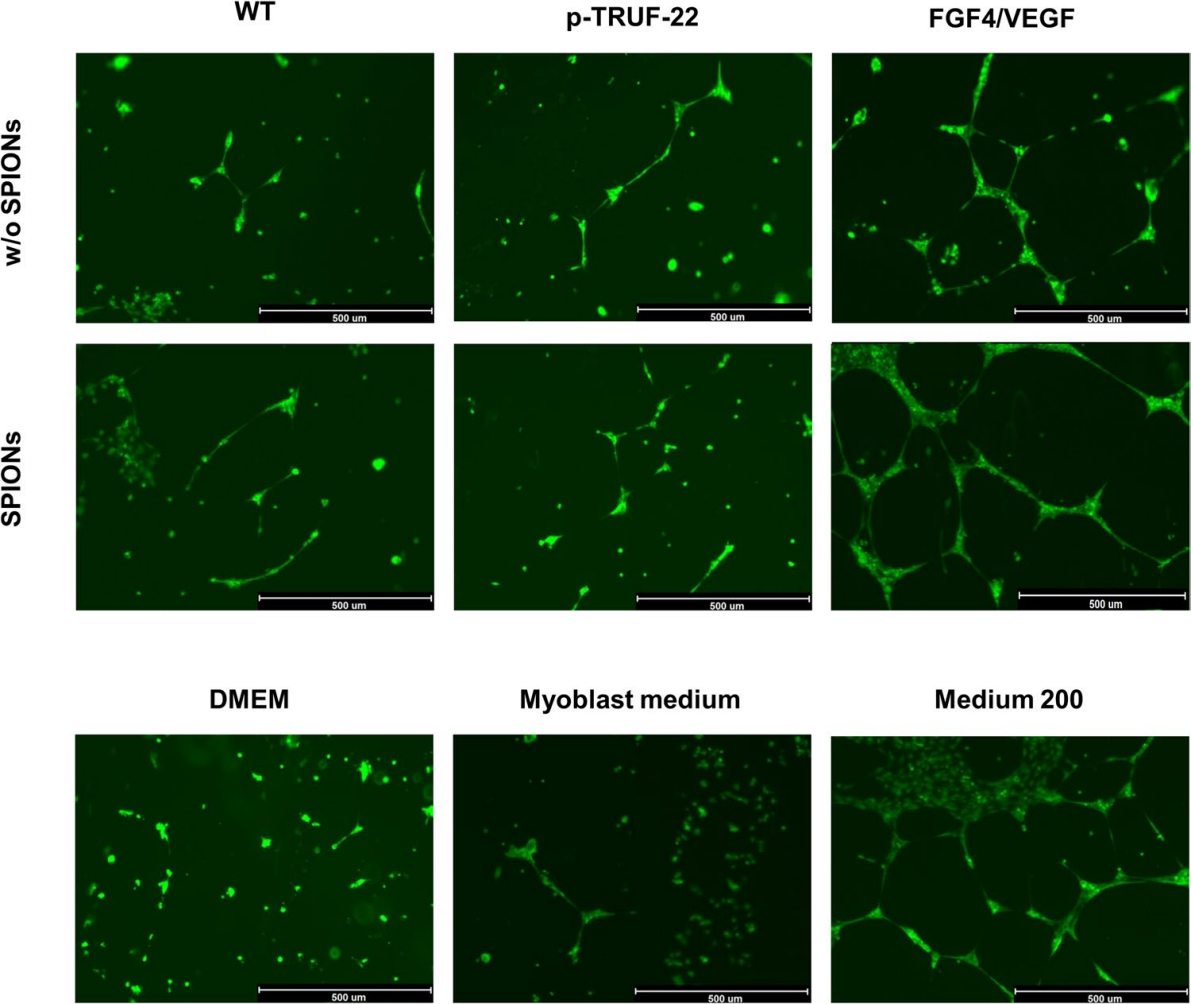
HUVEC angiogenesis test. To evaluate the pro-angiogenic properties of secreted proteins in the supernatants collected from myoblasts under study, the tested samples were transferred onto prepared HUVEC cells. Supernatants were collected from: WT – non transfected myoblasts; p-TRUF-22 – mock transfected myoblasts; FGF-4/VEGF – myoblasts transfected with complete bicistronic plasmid; w/o SPIONs – myoblasts non-labeled with obtained SPIONs; SPIONs – myoblast labeled with obtained SPIONs (0.025 mg/µL medium). Negative controls: DMEM and fresh myoblast medium. Positive control: medium 200 (with Large Vessel Endothelial Supplement). Capillaries were stained with calcein. Scale bars indicate 500 µm.

HUVEC angiogenesis assay did not show significant differences in the pro-angiogenic properties of secreted proteins in media collected from myoblasts labeled with Fe_3_O_4_-NPs versus myoblasts non-labeled with SPIONs in three different cell populations (WT, transfected with empty plasmid p-TRUF-22, transfected with bicistronic gene construct FGF4/VEGF) (Fig. 13). This suggests that using the obtained nanoparticles for transfected cells labeling we did not influence the biological functionality of transgenes.

### MRI and BLI

*In vitro* MR studies showed that the presence of SPION-labeled cells did not reduce T1 relaxation time at 7 T when compared to non-labeled cells (2830+/−32 ms vs. 2827+/−7 ms), but reduced T2 relaxation time (687+/−8 ms vs. 628+/−7 ms).

**Figure 14:**
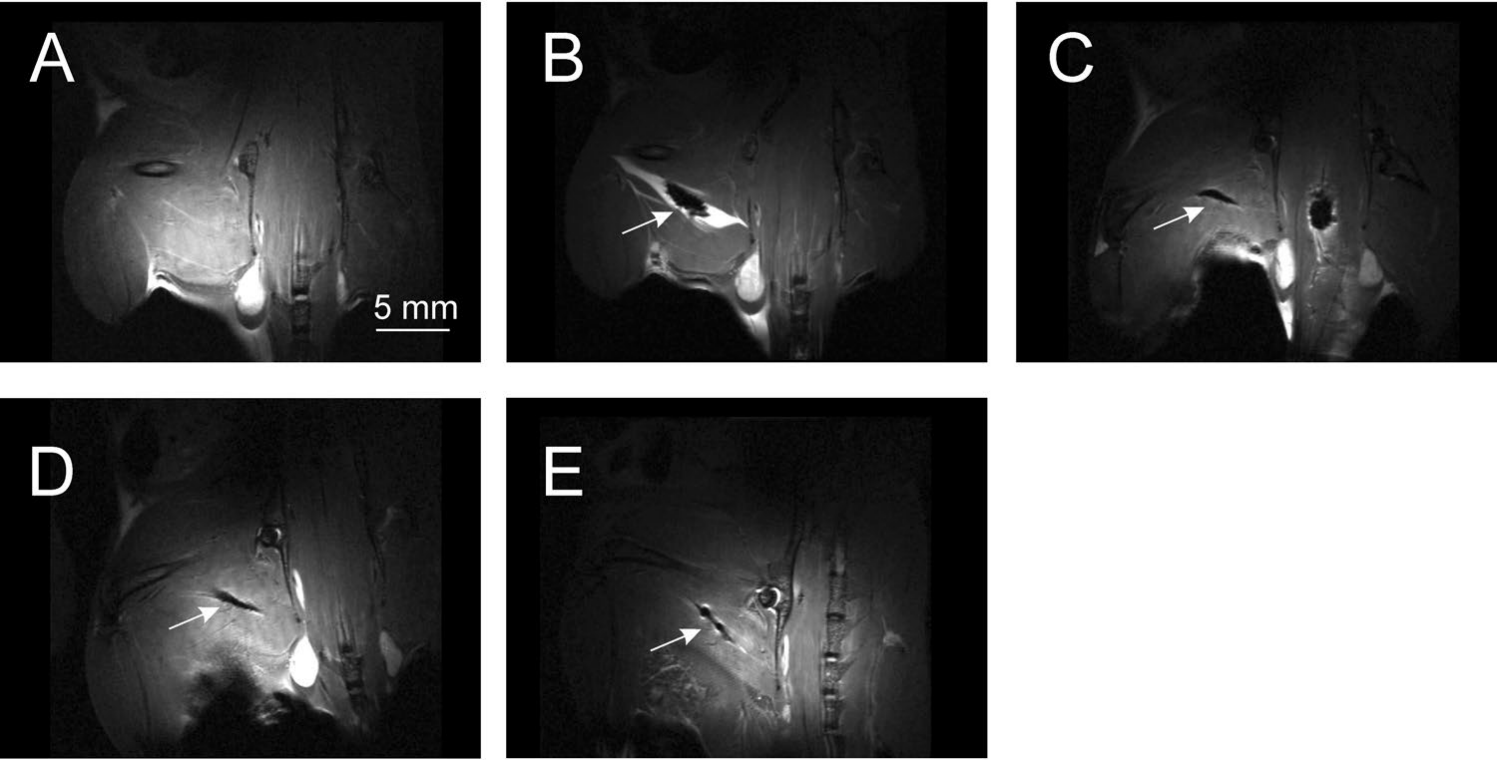
T2-weighted MR images of a mouse intramuscularly injected with SPION-labeled cells. Images were obtained before (**A**), immediately after the injection (**B**), one (**C**), two (**D**) and seven days (**E**) after the cell administration. Arrows point the hypointensive area at the site of injection. Bar represents 5 mm.

Injection of SPION-labeled cells to soleus muscle of the experimental animal resulted in clearly visible hypointensive zone corresponding to injection site on T2-weighted MR images. Images were obtained before, immediately after the injection, one, two and seven days after the cell administration (Fig. 14). This hypointensive area the site of injection was not visible before the injection but was observed in other timepoints, as long as seven days after the labeled cells administration.

**Figure 15:**
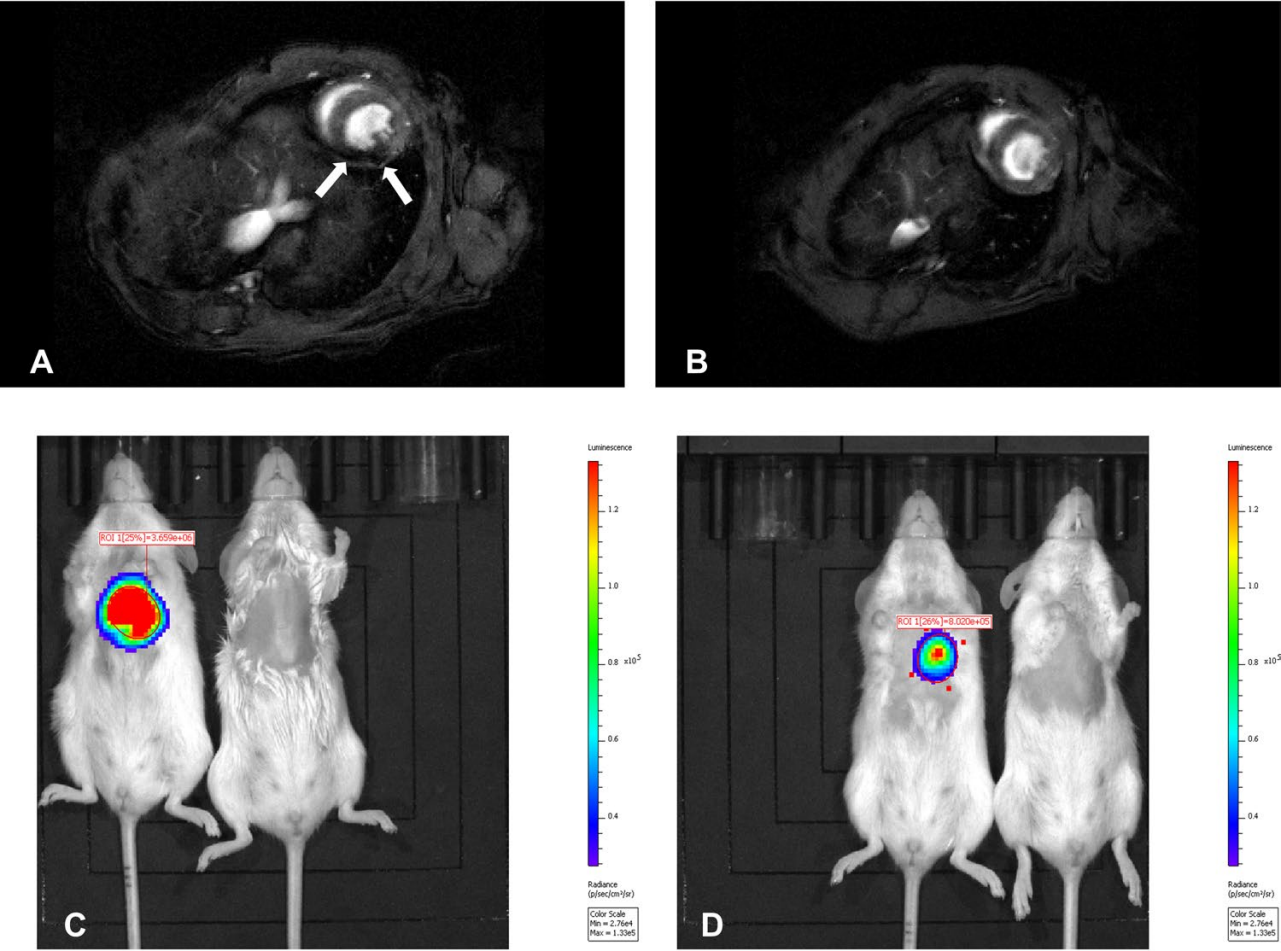
*In vivo* visualization of firefly luciferase-transduced myoblasts labeled with SPIONs. T2-weighted MR images were produced in mouse intracardially injected with SPION-labeled cells (**A**) versus control mouse (**B**). Arrows point out the hypointensive area at the site of injection. Bioluminescent imaging was acquired at 5 (**C**) and 12 (**D**) days after administration of cells and showed their distinct bioluminescent activity (left) as comparing to control mouse (right).

Intracardially administration of firefly luciferase-transduced and SPION-labeled myoblasts into the wall of the murine left heart ventricle also showed distinctly visible hypointensive area (Fig. 15A) comparing to the control mouse without labeled cells transplant (Fig. 15B). This visualized area corresponds to the site of cells administration. Moreover, *in vivo* bioluminescence imaging of transplanted myoblasts performed after MRI, showed clear signals from the site of cells administration at 5 and 12 days after myoblast implantation, respectively 3.659 × 10^6^ and 8.02 × 10^5^ of photon radiance (photons/sec/cm^2^/steradian). This means that MR images were obtained from viable SPION-labeled cells.

## Discussion

The aim of this study was to present an effective way of synthesis of biocompatible superparamagnetic iron oxide nanoparticles (SPIONs) for use as a labeling agent, and investigate *in vitro* their impact on human skeletal myoblasts, including both their phenotypic properties and molecular pathways. Utilizing SPIONs for direct labeling of cells, e.g. in stem cells therapy, could be the promising strategy for tracking transplanted stem cells. However, there was essential need to obtain adequate information about potential influence of SPIONs on basic cell functions.

To assess the influence of nanoparticles on myoblast cells, we first aimed to determine the cellular uptake of SPIONs by human skeletal myoblasts. For this purpose, we have performed qualitative Prussian Blue staining and then quantitative ICP-MS analysis. Especially, very precise mass spectrometry method showed prolific uptake of nanoparticles by cells with linear correlation (Supplementary Figure S1). Subsequently, we undertook the functional evaluation, comparing behavior of cells treated with iron oxide nanoparticles versus untreated ones. Although it is known that iron oxide can be processed *via* cellular iron metabolism pathway^33,34^ what makes it non-toxic to cells, there are some reports about negative effects of free Fe^3+^ ions (transition metals) on somatic cells^35–38^. We observed no differences in activity of reactive oxygen species between myoblasts treated with different concentrations of iron oxide nanoparticles and control wild type cells, what can suggest that obtained SPIONs do not promote oxidative stress in tested human myoblasts (Fig. 10A). There was also observed no cytotoxic effect of cells labeling with synthetized nanoparticles and no impact of obtained SPIONs on investigated myoblasts proliferation ability as well (Fig. 10B–E). Our studies showed no impact of labeling myoblasts with DMSA-coated SPIONs on their differentiation potential to myotubes comparing to non-labeled cells (Fig. 11A,B). Obtained results demonstrated only moderate changes in cell aging and cell apoptosis assays (Fig. 11C–E) concerning labeled myoblasts. Although we noticed significantly higher amounts of apoptotic cells within population of labeled myoblasts, this difference in our view should not have serious implications in *in vivo* applications. In cell aging assay we observed greater amounts of maturing cells in non-labeled myoblasts group and insignificant quantities of aged and young cells within myoblasts treated with iron oxide nanoparticles. These data let us to claim that DMSA-coated SPIONs have no significant effects on human skeletal myoblasts and indicate good biocompatibility of superparamagnetic iron oxide nanoparticles for potential *in vivo* application. Our results also correspond to previously reported studies about SPIONs and their impact studied on different cell lines^2,39,40^.

Potential influence of obtained DMSA-coated superparamagnetic iron oxide nanoparticles on genes expression profile in tested myoblast populations, was assessed by qRT-PCR experiments. We decided to investigate expression levels of *TFRC* and *FTL* – genes widely known as involved in cellular iron metabolism^1,3,41–43^ and genes reported to be involved in response to iron overload – *SIRT1* and *alpha-SMA*^43^. Moreover, we aimed to test whether utilizing DMSA as coating for SPIONs to increase their biocompatibility might also have an impact on genes expression, as it was reported in different cell lines for *EGR1*, *IFI27*, *GLI3* and *ID3*^1,3,41^.

We observed significant decrease of *TFRC* gene expression in myoblasts treated with iron oxide nanoparticles (Fig. 12). *TFRC* encodes transmembrane transferrin receptor, which plays a role in transfer of extracellular iron ions into the cell cytosol. This change in gene expression provides an information on mRNA level that tested myoblasts respond to SPIONs in their labeling environment. However, we did not observe any significant change in *FTL* expression between investigated labeled and non-labeled myoblast populations (Fig. 12). *FTL* encodes ferritin light chain, this protein plays a major role in intracellular iron storage in soluble and nontoxic state. Similar *FTL* expression levels in treated and untreated cells can mean that, although obvious response to iron oxide nanoparticles occurred, we did not overcome any critical levels of iron concentration influencing cell homeostasis. *FTL* expression level was also reported as biomarker of ageing^42,44^. Our qRT-PCR analysis of this gene expression remained in correlation with functional evaluation of tested myoblasts (differentiation), and we showed no significant differences in cell aging as measured in beta-galactosidase assay.

We checked expression levels of *SIRT1* and *alpha-SMA* since they were reported as involved in response to iron overload^43^. Sirtuin 1 (*SIRT1*) deacetylates wide spectrum of proteins and is involved in inflammatory responses, controls mitochondrial function and stress resistance^45,46^. It has been also reported a key role of *SIRT1*/*FOXO1* pathway in iron-overload induced myocardial injury in which *SIRT1* expression decreases^43^. In our studies we did not observe any decrease in expression of this gene, while we even have noticed a slight increase in SPIONs-treated myoblasts (Fig. 12). This fact may suggest that mechanisms of iron overload cascade have not been activated, but utilizing nanoparticle one could assume stimulation of some cellular protection pathways to preserve cell homeostasis.

Increased levels of alpha-smooth muscle actin (*alpha-SMA*) and others pro-fibrotic gene expression changes were observed in iron-induced myocardial fibrosis and cardiac dysfunction^43^. In our studies on possible SPIONs influence on gene expression profile of skeletal myoblasts, we observed decrease of alpha-SMA levels in labeled cells comparing to untreated ones (Fig. 12). Considering a potential application of labeled myoblasts in stem cell therapy of failing heart, this seems to be a promising feature.

Utilizing DMSA as coating for SPIONs to increase their biocompatibility was reported as a potential factor which may change genes expression profiles, like it was described for *EGR1*, *IFI27*, *GLI3* and *ID3* in different cell lines^1,3,41^. *EGR*1 was reported to be commonly repressed in two different human cell lines – THP-1 and HepG2 – after treatment with DMSA-SPIONs^41^. We observed statistically significant change of expression only in case of *EGR1*, which decreased in SPIONs-labeled myoblast population (Fig. 12) and this is in line of previous reports^1,41^. In case of *IFI*2*7* (reported to have increased expression in THP-1 and HepG2 cell lines after labeling with low and high doses of DMSA-SPIONs^41^), *GLI3* (the sole gene shared by THP-1, and HepG2 human cell lines and RAW264.7 murine cell line, of which expression was changed in all the three analyzed cell lines after treating them with DMSA-SPIONs^1^) and *ID3* (its decreased expression was proposed to be a potential toxicity biomarker for DMSA-SPION labeling^41^) we did not observe any significant changes in expression pattern of investigated myoblast population (Fig. 12), which can suggest that, although DMSA may have influence on some genes expression, our DMSA-coated nanoparticles did not disturb stem cell homeostasis.

In HUVEC angiogenesis assay we have shown not only that SPIONs-treated cells revealed similar pro-angiogenic properties as wild type cells (Fig. 13), but also that labeled myoblasts with iron oxide nanoparticles did not impact overexpressed protein function. This fact may also emphasize SPIONs to be safely used in stem cell therapy.

Further studies demonstrated that synthesized SPIONs showed excellent relaxation properties – altering effectively the T_2_ values with r_2_ exceeding 360 mM^−1^s^−1^- rendering them suitable for use as a sensitive MRI contrast agent in tissues with longer T_2_ characteristics (Fig. 8)^23,47^. These properties could be achieved due to narrow size distribution and proper crystal structure of obtained nanoparticles (Figs 2,3).

Finally, we have demonstrated that the proposed SPION-labeling was sufficient to visualize the cells *in vitro*, in experimental animals after intramuscular (Fig. 14) and intracardial administration (Fig. 15). Injection of SPION-labeled cells to soleus muscle and into the wall of left heart ventricle of the experimental animals resulted in clearly visible hypointensive zones corresponding to injection sites. Hypointensive area after intramuscular injection was observed as long as seven days after the labeled cells administration. Moreover, by labeling firefly luciferase-modified myoblasts, we demonstrated that cells visualized by MR are still viable *in situ*. This means that protocol labeling of human myoblasts with SPIONs developed was optimal while considering its utility for bioimaging of transplanted cells and their viability *in situ*. Indirectly, the combining of MRI and BLI methodology showed that myoblasts labeling with SPIONs did not impact overexpressed protein function.

In conclusion, in this study we showed that obtained DMSA-coated superparamagnetic iron oxide nanoparticles can be successfully utilized as a direct labeling agent for the cells which can make their *in vivo* tracking feasible. We demonstrated that SPIO itself did not disturb basic cellular functions and as such may render nanoparticles to be promising agents for potential stem cell therapy monitoring.

## Materials and Methods

All chemicals were bought from Sigma-Aldrich, unless otherwise stated. Ultrapure water was obtained from Hydrolab HL5 system with conductivity of 0.09 μS/mL.

### Synthesis of monodisperse iron oxide nanoparticles

#### Synthesis in organic phase

Iron oxide nanoparticles were synthesized according to thermal decomposition method. Briefly, 6 mmol iron (II) acetylacetate and 18 mmol of oleic acid were mixed in 50 mL of 1-octadecene in three-necked flask. The solution was stirred at 500 rpm and was saturated with nitrogen through a glass pipette. Then, the mixture was heated up to 220 °C and such temperature was held for 1 hour. Solution changed a colour from dark red, to dark bronze, then to yellow black and eventually turned black. After one hour, temperature increased to 320 °C and the reaction was refluxed for another hour. Condenser was introduced, otherwise the solution could evaporate. After cooling down, nanoparticles were washed with 100 mL mixture of toluene and 2-butanol (1:2 ratio), collected with permanent magnet and the procedure was repeated until the supernatant was transparent. Finally, nanoparticles were resuspended in 20 mL of chloroform.

#### Ligand exchange

In order to obtain biocompatible, hydrophilic nanoparticles ligand exchange procedure was performed to exchange the capping ligand from oleic acid to meso-2,3-dimercaptosuccinic acid (DMSA). The reaction contained two steps. In the first step, 50 mg of DMSA was dissolved in 15 mL dimethylsulfoxide (DMSO) and 100 mg of nanoparticles were diluted in 15 mL of chloroform. Solutions were mixed together and 50 μL of triethylamine was added as a reaction catalyst. Reaction was carried out at 60 °C for 6 hours (shaken vigorously) in a horizontal shaker. Nanoparticles were washed with ethanol, collected with permanent magnet and the procedure was repeated until the supernatant was transparent, and eventually nanoparticles were resuspended in 20 mL of ethanol. Then, the second step of reaction was carried out. Obtained solution was mixed with 50 mg of DMSA dissolved in 15 mL of DMSO and again 50 μL of triethylamine was added. The reaction conditions were the same as in the first step. Washing procedure was also similar, except that ultrapure water was used instead of ethanol. Finally, nanoparticles were resuspended in 10 mL of ultrapure water. For the use in *in vitro* tests, particles were sterile filtered with cellulose acetate syringe filters of two sizes: 0.45 μm and 0.2 μm, respectively.

### Nanoparticle characterization

#### Transmission Electron Microscopy (TEM)

The size and morphology was determined by Hitachi HT7700 Transmission Electron Microscope (TEM) at 120 kV for high resolution imaging (HR-TEM). For TEM, 10 μL samples were dropped on a carbon coated copper grids, mesh 200 l. HR-TEM images were analyzed with ImageJ software, using Fast Fourier Transform analysis in order to obtain crystal information.

#### Zeta Potential (ZP)

Zeta potential of DMSA coated nanoparticles was measured with Malvern Zetasizer Nano Z system, in a capillary cell. Measurements was carried out 3 times, with at least 20 counts in each one.

#### Fourier Transformed Infrared Spectroscopy (FT-IR)

Fourier Transformed Infrared (FTIR) measurements were conducted in order to confirm existence of magnetite and the process of ligand exchange on the nanoparticles surface. Samples were prepared in the form of powder mixed with KBr and pressed into pellets. Spectra were recorded on a spectrometer TENSOR 27 (Bruker Optics) in the region 4000–400 cm^−1^, with a resolution of 4 cm^−1^ and an accumulation of 256 scans.

#### Superconducting Quantum Interference Device (SQUID) and Nuclear Magnetic Resonance (NMR)

Hysteresis loops at 5 K and 300 K and ZFC-FC plots were obtained with SQUID MPMS XL Quantum Design system. The zero field-cooled (ZFC) and field-cooled (FC) magnetization curves were obtained using a standard protocol. After demagnetization at 300 K the system was cooled to 5 K without a magnetic field. Then, an external magnetic field of 100 Oe was applied, and the ZFC curve was recorded during heating to 300 K. The FC curve was measured during cooling from 300 K down to 5 K in the same external magnetic field. Magnetic relaxivity was investigated with Bruker Ultrashield NMR at 300 MHz frequency. For determination of *T*_1_ and *T*_*2*_ relaxation times, inversion recovery measurements at 37 °C and spin echo measurements at 37 °C were conducted, respectively.

### Myoblast isolation and *in vitro* culture

The protocol of tissue collection was approved by the Local Bioethical Committee, Poznan University of Medical Sciences and the written consent was obtained from each study participant. At the same time we should like to reassure that all the experiments performed with human materials were in accordance to the relevant guidelines and regulations.

Human myoblast cells were isolated from fragments of skeletal muscle tissue harvested from patients as a tissue waste after the reconstruction of an anterior cruciate ligament (ACL). After removing the remnants of adipose tissue from the obtained samples, muscle fragments were mechanically dissected and subjected to digestion with 0.02% collagenase solution (Sigma-Aldrich, St. Louis, MO, USA) in water bath (33 °C) with orbital shaking for 45 minutes. Next, the cell suspensions were filtered through an 80 μm mesh, centrifuged and seeded in 25 cm^2^ culture flasks (Becton Dickinson, Franklin Lakes, NJ, USA) coated with 0.1% gelatine (Sigma-Aldrich, St. Louis, MO, USA). To obtain the human primary myoblast cultures the modified ‘preplate’ method was used^48^. The purity of myoblast population was evaluated by the presence CD56 surface marker as previously described^49^. Immunostaining for desmin, the muscle cell marker, was also performed according to protocol earlier described^20^. Cells were cultured in standard Dulbecco’s modified Eagle’s medium (DMEM) containing 4.5 g/L of glucose, supplemented with 20% fetal bovine serum (Lonza Group, Base, Switzerland), 1% antibiotic mixture - Pen/Strep/Amphotericin B (Lonza Group, Base, Switzerland), 1% Ultraglutamine (L-Alanylo-L-Glutamina, Lonza Group, Base, Switzerland), 1% Chicken Embryo Extract (CEE, SeraLab, West Sussex, UK) and fibroblast growth factor (bFGF, Sigma-Aldrich, St. Louis, MO, USA) to sustain *in vitro* proliferation. All *in vitro* cultures were carried out in medium at 37 °C and 5% CO_2_ concentration in standard T-flasks (75 cm^2^). The medium was changed every 48 hours and the cells were split after they reached 75% confluence using 0.25% trypsin (Lonza Group, Base, Switzerland).

### Iron oxide-nanoparticles cell labeling and SPIONs uptake evaluation

In order to qualitatively evaluate the SPION uptake, Prussian blue staining was performed. Solution of iron oxide nanoparticles (Fe_3_O_4_-NPs) coated with DMSA was diluted in supplemented DMEM medium to obtain desired concentrations of SPIONs: 0.1 mg/mL, 0.05 mg/mL, 0.025 mg/mL, 0.0125 mg/mL, 0.00625 mg/mL. Cells were cultured in as-prepared media with Fe_3_O_4_-NPs for 24 hours, then washed with PBS (Lonza Group, Basel, Switzerland) to remove residual SPIONs and the fresh medium (without Fe_3_O_4_-NPs) was added. All the tests were carried out 48 hours after the administration of media containing SPIONs to the cells, unless otherwise stated. To visualize the uptake of Fe_3_O_4_-NPs, Prussian Blue staining was also performed 24 hours after myoblasts labeling with SPIONs. For this purpose, cells were washed with PBS and following fixed with 4% paraformaldehyde. Subsequently, the cells were washed with deionized water and stained using Iron Staining Kit (Sigma-Aldrich, St. Louis, USA). Afterwards, the cells were washed again with deionized water and photographs were taken using light microscope.

In order to quantitatively determine the uptake of nanoparticles, the total cellular iron content was measured with inductively coupled plasma mass spectrometry (ICP-MS). The analysis was performed on a Perkin-Elmer Nexion 300D quadrupole mass spectrometer. Cell labeling procedure was the same as for the Prussian Blue staining. Similarly, final concentrations of SPIONs in culture media were 0.1 mg/mL, 0.05 mg/mL, 0.025 mg/mL, 0.0125 mg/mL, 0.00625 mg/mL. Then, we followed a modified procedure previously described^50^. Briefly, cell pellets of SPIONs-treated myoblasts (4 × 10^5^ cells) were collected and homogenized in 100 µL of 10% SDS. Then, the cell lysates were frozen for 24 hours and, after thawing, sonicated for 1 hour. 25 µL of cell lysates (1 × 10^5^ cells) were dissolved in 125 µL nitric acid (POCH, 65%, pure) and shaken in 80 °C for 2 hours to remove organic compounds. Then, the samples were diluted 10 times with ultrapure water and were taken for measurements. For comparison, SPION “standard” solutions with the investigated nanoparticle concentrations were prepared in untreated cell lysates (4 × 10^5^ cells in 100 µL of 10% SDS) and prepared for measurement as above. Iron content was determined based on the standard curve prepared with a multi element standard solution for ICP-MS in a 1, 10, 100, 1000 ppb range.

### Reactive Oxygen Species (ROS) test

The development of reactive oxygen species (ROS) in tested cell populations was detected using DCFDA Cellular ROS Detection Assay Kit (Abcam, Cambridge, UK) according to producer’s manual. Investigated cells were seeded at density of approximately 6,000 cells per well on 96-well microplate (flat and clear bottom, dark side), 10 wells per group. Microplate was read using up-read plate reader GLOMAX^®^ Multi Detection System (Promega, Madison, WI, USA) with excitation/emission wavelengths filter: 490/510–570 nm. The relative fluorescence of each tested myoblast sample was normalized with reference to fluorescence of wild type myoblast without any reagent. Then average relative fluorescence of non-labeled myoblasts was equated to 100% and the remaining values were calculated proportionally.

### Cytotoxicity and cells proliferation

Potential cytotoxicity of synthesized SPIONs and their impact on cells proliferation were investigated using CyQUANT^®^ Direct Cell Proliferation Assay Kit (Invitrogen, Carlsbad, CA, USA) following the manufacture’s protocol. Human skeletal myoblasts were plated at density of 20,000 cells per well on 96-well microplate (flat and clear bottom, dark side), 10 wells per group for cytotoxicity assay. For cell proliferation assay, myoblasts were plated at the density of 10,000 cells per well on 96-well microplate (flat and clear bottom, dark side), 10 wells per group. The following day, the culture medium from each well was replaced with 200 μL of fresh medium containing Fe_3_O_4_-NPs at examined concentrations or with 200 μL of fresh medium containing 500 μM H_2_O_2_ as negative control. Meanwhile, the cells incubated with culture media without nanoparticles were considered as untreated control. After 24 hours of incubation at 37 °C, cells were washed with PBS (Lonza Group, Base, Switzerland) and the fresh complete medium was added. Potential cytotoxicity of SPIONs was tested 24 hours after treatment cells with nanoparticles or 500 μM H_2_O_2_ and cells proliferation was investigated 72 hours after exposure for potential stressors. Microplates were read using bottom-read plate reader Cytation™ 3 Cell Imaging Multi-Mode Reader (BioTek Instruments, Inc., VT, USA) with excitation/emission wavelengths filter: 480/535 nm and 7 × 7 matrix size of single well area scan. Obtained fluorescence values in each investigated myoblast group were averaged. Recalculated as percentage averaged fluorescence values of non-labeled cells were equated to 100% and the rest of the values were calculated proportionally to them.

### Myogenic differentiation potential

Myogenic differentiation was induced by providing to *in vitro* cultures (at almost 100% confluence) medium containing 2% horse serum (Sigma-Aldrich, St. Louis, USA) instead of 20% FBS and growth factors. Cells were *in vitro* cultured for 7 days in differentiation medium, then multinucleated myotubes were observed. To estimate the myogenic potential of tested cell populations – wild type myoblasts and Fe_3_O_4_-NPs-labeled myoblasts – the percentage value of nuclei number present in differentiated myotubes to the number of nuclei in undifferentiated cells was compared. The differentiated cell populations were fixed in freezing methanol:acetic acid (3:1) for 15 minutes, washed with 1 × PBS, stained using a 10% Giemsa solution (Merck, Darmstadt, Germany) for 30 minutes and washed in distilled water containing 0.005% acetic acid. Photographs were taken using light microscope and at least 500 nuclei were counted in analyzed cell populations.

### Cytochemical staining with SA-beta-galactosidase to assess cell aging

To analyze cellular senescence, cytochemical staining with SA-beta-galactosidase followed. The analysis was performed using Cell Aging Senescence Detection Kit (BioVision, Milpitas, CA, USA), according to manufacturer’s instruction. Cells were fixed in fixative solution (provided by manufacturer) and left at 37 °C in X-Gal staining solution for overnight incubation. Then, the cells were counterstained with eosin and evaluated under the light microscope. Three cell populations were evaluated: 1 - SA-beta-galactosidase negative; 2 - moderately stained; 3 - expressing SA-beta-galactosidase staining. Cells were counted in respective samples.

### Apoptosis evaluation

The process of apoptosis was detected in tested cell populations (Fe_3_O_4_-NPs-labeled and wild type) using Annexin V-FITC Kit (Beckman Coulter). Every step of analysis was performed according to producer’s manual. The cells were resuspended in mixture with FITC-conjugated Annexin V and Propidium Iodide. Samples were incubated for 15 minutes in darkness. Then, samples were analyzed on the Cell Lab Quanta cytometer (Beckmann-Coulter).

### Real-time PCR – gene expression evaluation

Total RNA from wild type and Fe_3_O_4_-NPs-treated cells was isolated using AllPrep DNA/RNA/Protein Mini Kit (Qiagen, Hilden, Germany) according to manufacturer’s protocol. To avoid DNA contamination, samples were treated with TURBO DNA-Free^TM^ Kit (Invitrogen, Carlsbad, CA, USA). The yield and quality of isolated RNA were analyzed using a Nanodrop 2000 (Thermo Scientific, Waltham, MA, USA) and 1.5% agarose gel electrophoresis. Subsequently, a 3 μg aliquot of isolated RNA was reversely transcribed using SuperScript IV Reverse Transcriptase (Invitrogen, Carlsbad, CA, USA). The resultant cDNA was diluted (4 × ) and evaluated by regular PCR reaction with primers for β-actin gene. PCR reaction efficiency for each applied primer pair was obtained from the standard curve. Serial dilutions 10^2^–10^8^ copies/μL of amplicon for every assessed gene were prepared. The primers for specific genes were obtained from PrimerBank^51–53^ (https://pga.mgh.harvard.edu/primerbank/) or designed and purchased from Genomed (Warsaw, Poland) and have been listed in Table 1.

**Table 1:** Primer sequences for genes under study.

GENE ID	PRIMER	SEQUENCE	GENE BANK ID	Amplicon size (bp)
*TFRC*	Forward	ACCATTGTCATATACCCGGTTCA	189458816c1	219
	Reverse	CAATAGCCCAAGTAGCCAATCAT		
*FTL*	Forward	CAGCCTGGTCAATTTGTACCT	56682960c1	114
	Reverse	GCCAATTCGCGGAAGAAGTG		
*SIRT1*	Forward	TAGCCTTGTCAGATAAGGAAGGA	215982797c1	160
	Reverse	ACAGCTTCACAGTCAACTTTGT		
*alpha-SMA*	Forward	AAAAGACAGCTACGTGGGTGA	213688378c1	76
	Reverse	GCCATGTTCTATCGGGTACTTC		
*EGR1*	Forward	ACCGCAGAGTCTTTTCCTGA	designed	79
	Reverse	GAGTGGTTTGGCTGGGGTAA		
*IFI27*	Forward	TGCTCTCACCTCATCAGCAGT	194272170c1	115
	Reverse	CACAACTCCTCCAATCACAACT		
*GLI3*	Forward	TGGTTACATGGAGCCCCACTA	195947346c2	116
	Reverse	GAATCGGAGATGGATCGTAATGG		
*ID3*	Forward	GAGAGGCACTCAGCTTAGCC	345199309c1	170
	Reverse	TCCTTTTGTCGTTGGAGATGAC		
*ACTB*	Forward	CTTCCTGGGCATGGAGTCC	designed	192
	Reverse	ATCTTGATCTTCATTGTGCTG		
*GAPDH*	Forward	GCTCTCTGCTCCTCCTGTTC	designed	112
	Reverse	ACCAAATCCGTTGACTCCGA		
*HPRT1*	Forward	CCTGGCGTCGTGATTAGTGA	designed	167
	Reverse	TGATGGCCTCCCATCTCCTT		

Abbreviations: *TFRC* – transferrin receptor 1; *FTL* – ferritin light chain; *SIRT1* – sirtuin 1; *αSMA* – alpha smooth muscle actin; *EGR1* – early growth response 1; *IFI27* – interferon alpha inducible protein 27; *GLI3* – GLI family zinc finger 3; *ID3* – inhibitor of DNA binding 3; *ACTB* – β-actin; *GAPDH* – glyceraldehyde-3-phosphate dehydrogenase; *HPRT1* – hypoxanthine phosphoribosyltransferase 1.

The qPCR experiments were performed using iCycler detecting system (BioRad, Hercules, CA, USA) and iQ^™^ SYBR^®^ Green Supermix Reagent (BioRad, Hercules, CA, USA). Single reaction volume sample was 10 μL and contained: 2 μL of cDNA, 5 μL of 2 × iQ^™^ SYBR^®^ Green Supermix Reagent (BioRad, Hercules, CA, USA) and 1 μL of each 4 μM primer. The following conditions were used: 95 °C for 1 minute, 45 cycles at 95 °C for 20 seconds, 60 °C for 20 seconds and 72 °C for 20 seconds. The relative expression level of each studied transcript (*TFRC* – transferrin receptor 1, *FTL* – ferritin light chain, *SIRT1* – sirtuin 1, *αSMA* – alpha smooth muscle actin, *EGR1* – early growth response 1, *IFI27* – interferon alpha inducible protein 27, *GLI3* – GLI family zinc finger 3, *ID3* – inhibitor of DNA binding 3) was normalized with reference to three housekeeping genes (*ACTB* – β-actin, *GAPDH* – glyceraldehyde-3-phosphate dehydrogenase, *HPRT1* – hypoxanthine phosphoribosyltransferase 1) according to Vandesompele *et al*.^54^.

### HUVEC angiogenesis test

To determine the pro-angiogenic properties of proteins expressed in transfected myoblast populations, the HUVEC angiogenesis test was performed. Human myoblasts were transfected *via* electroporation with p-TRUF-22 and FGF-4/VEGF plasmids as previously described^55^ and then labeled with Fe_3_O_4_-NPs. HUVECs were cultured (no further than to 4th passage) to reach about 80–90% confluency using Medium 200 supplemented with Large Vessel Endothelial Cells Supplement - LVES (Gibco, Carlsbad, USA). In the next step, cells were trypsinized, counted and seeded at density of approximately 25,000 cells per cm^2^ on 24-well plate coated with Geltrex^®^ Matrix. HUVECs were covered with the supernatants collected from the myoblast culture samples, in order to determine the functional properties of secreted proteins. DMEM and standard myoblast medium were used as a negative controls and Medium 200 was used as a positive control. Then, HUVECs were incubated with supernatants harvested from myoblasts and cultured for 14–18 hours. After this time newly formed capillaries were stained with 2 μg/mL of Calcein-AM (Invitrogen, Carlsbad, USA).

### Magnetic resonance imaging *in vitro*

SPION-labeled cells and non-labeled cells were scanned in 7 T BrukerBiospec tomograph (70/30 USR, Bruker Biospin, Ettlingen, Germany). Structural MR images were acquired with T2-weighted TurboRARE-2D sequence (TR = 2000 ms, TEeff = 20 ms, slice thickness 0.6 mm, Scan time ~ 11 min). Set of images to calculate T1 relaxation time values were performed using 2D Saturation Recovery Spin Echo Sequence (TRs 300, 600, 1000, 2000, 5000, 10000, 18000 ms, TEeff = 13 ms, scan time ~ 31 min). T2 relaxation time calculation was based on Multi Slice Multi Echo sequence (TR 5000 ms, TEs [15… 480] ms, echo spacing 15 ms, number of echoes = 32, scan time ~ 8 min).

### Magnetic resonance and bioluminescence imaging *in vivo*

Animal experiments were performed in accordance with the EU Directive 2010/63/EU for animal experiments and respective local regulations. Animal research followed also internationally accepted guidance for the care and use of laboratory animals, including the National Institute of Public Health – National Institute of Hygiene (NIPH – NIH) guidelines, and was approved by the respective Local Ethical Committee for Animal Experimentation, Poznan University of Life Sciences. We should like also to confirm that all animal experiments were performed in accordance with relevant guidelines and regulations.

BALB/c mouse (MMRC in-house colony) was anesthetized with 1.5–2% isoflurane (Baxter, Deerfield, IL, USA) in oxygen, and positioned with the head placed prone in the MR compatible animal bed. A receive-only loop coil (10 mm internal diameter; used in combination with transmit cylindrical radiofrequency volume coil, 8.6 cm inner diameter) was placed over soleus muscle of the mouse and the animal was placed in 7 T BrukerBiospec tomograph (70/30 USR, Bruker Biospin, Ettlingen, Germany). Structural MR images were then acquired with T2-weighted TurboRARE-2D sequence (TR = 1400 ms, TEeff = 30 ms, NA = 5, spatial resolution = 0.117 mm × 0.117 mm, slice thickness 0.6 mm, Scan time ~ 8 min). Immediately after the scanning, the animal was removed from the scanner and intramuscularly injected with SPION-labeled cells. After the cells administration, the animal was immediately subjected to the same imaging procedure. MR imaging was also repeated after 1, 2 and 7 days.

Firefly luciferase-modified and SPION-labeled myoblasts (1.5 × 10^6^) were transplanted into wall of the left heart ventricle of NOD-SCID mouse, as previously described^20^. Lentiviral transfer vector pLV-EF1a-Fluc-T2A-mCherry-PGK-Neo was designed and obtained from VectorBuilder (www.vectorbuilder.com). Lentivirus production in HEK293T cells including transfer vector DNA, psPAX2 packaging and pMD2.G envelope plasmid DNA (ratio of 4:3:1, respectively) and lentiviral transduction of myoblasts were performed according to described protocol^56^ with minor modifications. Modified myoblasts were selected by culturing in medium with 1.5 mg/mL of G418 (Thermo Scientific, Waltham, MA, USA) and labeled with SPIONs (0.025 mg/mL). Magnetic resonance imaging *in vivo* of transplanted cells was performed on the next day after myoblasts administration into wall of the left heart ventricle with reference to control NOD-SCID mouse. *In vivo* bioluminescence imaging (BLI) of administered cells was performed at day 5 and 12 after cell transplantation. Investigated and control mice were anesthetized with 2% isoflurane in oxygen and injected intraperitoneally with XenoLight D-Luciferin (PerkinElmer, Waltham, MA, USA) according to producer’s manual. The mice were then imaged under anesthesia (2% isoflurane in oxygen) using *in vivo* bioluminescence imaging system (IVIS^®^ Lumina LT Series III, PerkinElmer, Waltham, MA, USA). Imaging parameters included field of 12.5 cm, exposure time of 2 minutes, number of binning 8, F/stop of 1 and emission filter open. Consecutive 2-minutes frames were acquired until the maximum signal was reached. The signal intensity were represented by radiance (p/sec/cm2/sr) from a circular auto ROI measurement.

### Statistical analysis

Each experiment was performed at least three times and each experimental group was repeated in triplicate. The quantitative PCR and apoptosis evaluation results were assessed using the t-test with Welsh’s correction. The ROS detection, cytotoxicity, cells proliferation and SPIONs uptake evaluation assays results were calculated using ANOVA together with Tukey’s multiple comparison test. The differentiation potential and cell aging assay results were analyzed using two-way ANOVA and Bonferroni’s multiple comparison test. All the results (if not otherwise stated) are presented as mean ± SEM.

## Electronic supplementary material

Supplemmentary Information
