# Supplementary material for: Potential use of superparamagnetic iron oxide nanoparticles for in vitro and in vivo bioimaging of human myoblasts
Source: Sci Rep. 2018 Feb 27;8:3682. doi: 10.1038/s41598-018-22018-0 (PMC5829264; doi:10.1038/s41598-018-22018-0)

Potential use of superparamagnetic iron oxide nanoparticles for *in vitro* and *in vivo* bioimaging of human myoblasts.

Kamil R. Wierzbinski[1#], Tomasz Szymanski[1,2,3#], Natalia Rozwadowska[1], Jakub D. Rybka[2,3], Agnieszka Zimna[1], Tomasz Zalewski[4], Karolina Nowicka-Bauer[1], Agnieszka Malcher[1], Magdalena Nowaczyk[1], Michal Krupinski[5], Michal Fiedorowicz[6], Piotr Bogorodzki[6], Pawel Grieb[6], Michal Giersig[2,3,7], Maciej K. Kurpisz[1*]

[1] Institute of Human Genetics Polish Academy of Sciences, Poznan, Poland

[2] Faculty of Chemistry, Adam Mickiewicz University, Poznan, Poland

[3] Wielkopolska Centre of Advanced Technologies, Adam Mickiewicz University, Poznan, Poland

[4] NanoBioMedical Centre, Adam Mickiewicz University, Poznan, Poland

[5] The Henryk Niewodniczanski Institute, Institute of Nuclear Physics Polish Academy of Sciences, Cracow, Poland

[6] Mossakowski Medical Research Centre, Polish Academy of Sciences, Warsaw, Poland

[7] Institute of Experimental Physics, Freie Universität Berlin, Berlin, Germany

[#] Both authors equally contributed to the study

[*]Corresponding Author

Maciej K. Kurpisz MD PhD,

Institute of Human Genetics,

Polish Academy of Sciences, Poznan, Poland

e-mail: kurpimac@man.poznan.pl

Supplementary Figure S1: Diagrams representing results of ICP-MS analysis. **A-B** – iron concentrations in standard solutions of SPIONs in cell lysates. **C-D** – iron concentrations in SPION-treated cell lysates. **E-F** – calculated amounts of iron per single cell. Abbreviations: ICP-MS – inductively coupled plasma mass spectrometry. Asterisks indicate statistical significance (** $p < 0.01$; *** $p < 0.001$).

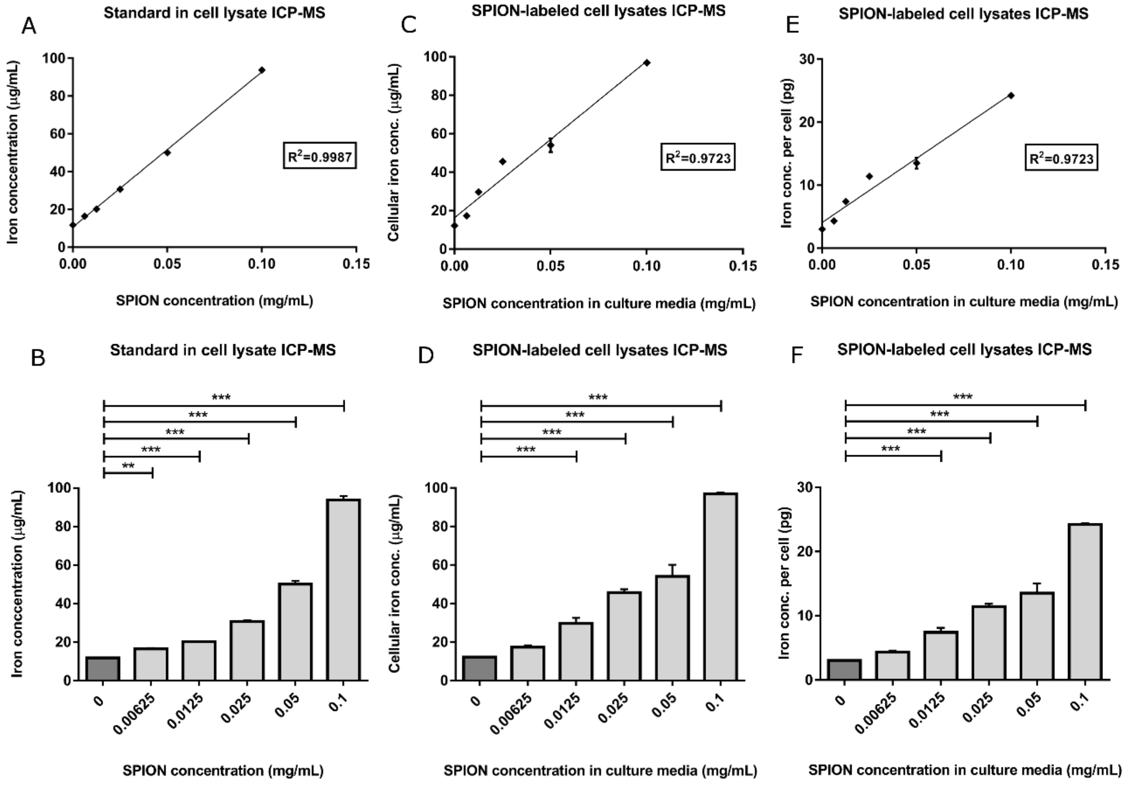

Supplement: Supplementary file 1 — Supplemmentary Information [file 41598_2018_22018_MOESM1_ESM.pdf]
